# The UK Healthy Universities Self-Review Tool: Whole System Impact

**DOI:** 10.1093/heapro/daw099

**Published:** 2016-12-21

**Authors:** Mark Dooris, Alan Farrier, Sharon Doherty, Maxine Holt, Robert Monk, Susan Powell

**Affiliations:** 1 Healthy and Sustainable Settings Unit, University of Central Lancashire, Brook Building, Victoria Street, Preston PR12HE, UK; 2 Faculty of Health, Psychology & Social Care, Manchester Metropolitan University, Brooks Building, 53 Bonsall Street, Manchester M156GX, UK

**Keywords:** settings, health promotion, higher education, universities, assessment

## Abstract

Over recent years, there has been growing interest in Healthy Universities, evidenced by an increased number of national networks and the participation of 375 participants from over 30 countries in the 2015 International Conference on Health Promoting Universities and Colleges, which also saw the launch of the Okanagan Charter. This paper reports on research exploring the use and impact of the UK Healthy Universities Network’s self review tool, specifically examining whether this has supported universities to understand and embed a whole system approach. The research study comprised two stages, the first using an online questionnaire and the second using focus groups. The findings revealed a wide range of perspectives under five overarching themes: motivations; process; outcomes/benefits; challenges/suggested improvements; and future use. In summary, the self review tool was extremely valuable and, when engaged with fully, offered significant benefits to universities seeking to improve the health and wellbeing of their communities. These benefits were felt by institutions at different stages in the journey and spanned outcome and process dimensions: not only did the tool offer an engaging and user-friendly means of undertaking internal benchmarking, generating an easy-to-understand report summarizing strengths and weaknesses; it also proved useful in building understanding of the whole system Healthy Universities approach and served as a catalyst to effective cross-university and cross-sectoral partnership working. Additionally, areas for potential enhancement were identified, offering opportunities to increase the tool’s utility further whilst engaging actively in the development of a global movement for Healthy Universities.

## INTRODUCTION

Globally, universities are important organisations for health promotion, not only as contexts and vehicles for enhancing wellbeing, but also as partners in multi-sectoral health improvement and contributors to societal change (Dooris *et al.*, 2012). Within the UK, universities have long been contexts in which specific health-related projects are delivered, prompted by concern about staff and student wellbeing (Dooris and Doherty, 2010a). Reflecting the success of other settings initiatives, higher education institutions (HEIs) have become increasingly interested in using a strategic ‘whole university approach’ which seeks to join-up health topics and cross-cutting issues in a way that will involve the whole university community (Dooris and Doherty, 2010b). This draws on learning from the healthy settings model of health promotion, which recognises that “health is created and lived by people within the settings of their everyday life; where they learn, work, play and love” (WHO, 1986) and is characterized by an ecological model of health, a systems perspective and a whole system focus – concerned to embed health within the culture, ethos structures and routine life of settings (Dooris, 2013).

It has been argued that a Healthy University “aspires to create a learning environment and organisational culture that enhances the health, wellbeing and sustainability of its community and enables people to achieve their full potential” (Dooris *et al.*, 2010). Alongside this, it acknowledges its role in ‘future shaping’ students and staff as they clarify values, grow intellectually and develop capabilities that can enhance current and future citizenship. Underpinned by principles such as partnership, equity, participation and empowerment, the Healthy Universities approach aims to be proactive in planning for health and achieving impacts and long-term outcomes in relation to both public health and core business agendas (Dooris *et al.*, 2012), through:

- creating healthy and sustainable learning, working and living environments for students, staff and visitors
- integrating health and sustainability as multi-disciplinary cross-cutting themes in curricula, research and knowledge exchange
- contributing to the health, wellbeing and sustainability of local, regional, national and global communities.

The UK Healthy Universities Network (undated) emerged out of the English Network, established in 2006. In consultation with HEIs, a website was developed incorporating a toolkit comprising guidance packages, case studies and a self review tool (SRT) to support healthy university work. Since 2012, the SRT has provided a mechanism for HEIs to review their progress in embedding a whole system approach to health and wellbeing within their core business and culture. It comprises an online questionnaire structured under five ‘process’ headings, each with a number of sub-headings under which questions are asked (see Table 1). The SRT was developed through widespread consultation with Network members and the headings and sub-headings reflect the centrality of organisation development, change management and creating supportive environments within the healthy settings approach (Grossman and Scala, 1993; WHO, 1986), as well as the importance of areas such as leadership, planning, implementation, stakeholder engagement, and communication within core internationally-agreed health promotion competencies and professional standards (Dempsey *et al.*, 2011; Speller *et al.*, 2011). For each question, respondents choose from one of four answers: ‘yes, we are there’, ‘working on this currently’, ‘thinking about it’ and ‘no, not at all’. Once a university has completed the questionnaire, a ‘traffic light’ report is generated, showing progress and highlighting strengths and weaknesses. The SRT is confidential and not designed to provide comparative benchmarking. Rather, it offers a self–assessment process, allowing individual HEIs to generate their own evidence and determine their own priorities.

**Table 1:** UK Healthy Universities Network Self Review Tool – Questionnaire Headings and Sub-Headings

1.	Leadership and Governance
	a) Corporate Engagement and Responsibility
	b) Strategic Planning and Implementation
	c) Stakeholder Engagement
2.	Service Provision
	a) Health Services
	b) Wellbeing and Support Services
3.	Facilities and Environment
	a) Campus and Buildings
	b) Food
	c) Travel
	d) Physical Activity, Recreational and Social Facilities
	e) Accommodation
4.	Communication, Information and Marketing
	a) Communication
	b) Information
	c) Marketing
5.	Academic, Personal, Social and Professional Development
	a) Curriculum
	b) Research, Enterprise and Knowledge Transfer
	c) Professional Development

## AIMS AND OBJECTIVES

This study was undertaken by a team from University of Central Lancashire and Manchester Metropolitan University, with a view to examining whether the SRT has supported universities to understand and embed a whole system approach.

The aims were to:

- Scope the use and impact of the SRT
- Inform future developments.

The objectives were to:

- Collate data on use of the SRT in terms of motivation, context, timing and process
- Identify benefits
- Identify issues and challenges
- Generate recommendations to inform future development and use.

## METHODS

The study received ethical approval from both universities involved in the research. It comprised two stages:

### Stage One

An online questionnaire was used to examine the use of the SRT in terms of process, perceived user-friendliness, outcomes/benefits, negative consequences and suggested modifications. All members of the UK Healthy Universities Network and non-members having signed up online to use the SRT [a total of 253 people from 84 institutions] were invited to complete the questionnaire. The email highlighted that, for many HEIs, the invitation was being sent to more than one person and that recipients may wish to liaise with each other. Three reminder emails were sent before closing the questionnaire, which was completed by 25 people from 19 institutions (15 from the UK, 2 from Canada, 1 from the USA and 1 from Australia), a response rate of 10% for individuals and 23% for institutions.

### Stage Two

Focus groups (Kitzinger, 1994) were used to explore the experiences of HEIs which had used the online SRT. A total of 73 HEIs worldwide had registered online to use the tool and, of these, 28 had actually used it. As the tool was designed for use within a UK context, it was decided that an invitation to participate in this second stage of the study should be sent only to the UK HEIs that were recorded as having completed the online SRT – a total of 17 (out of 47 UK institutions that had registered to use the tool). Of these, six) offered to take part. Focus groups were then undertaken using ‘Skype’ with small groups of individuals from five of these universities (as one was unable to participate). The individuals were selected by the Healthy University co-ordinator or other lead contact at each participating university, drawn from those stakeholders involved in their SRT process. A semi-structured focus group schedule was used – exploring motivation, process, content/structure, expectations, outcomes, experience and relationship to a whole system approach. Focus groups were facilitated by members of the research team and audio-recorded.

Questionnaire data were analysed using an online tool, *Qualtrics* and further interrogated by research team members to identify key emerging themes. Focus group data were transcribed and subjected to thematic analysis, involving: familiarising with the data; generating initial codes; searching for themes; reviewing; defining and naming themes; and producing a report (Braun and Clarke, 2006).

## FINDINGS

### Overview

Within Stage One of the study, 19 universities completed the questionnaire. Eleven of these had used the SRT, two were planning to use it, three had not used it and three did not respond to the question. Of the eleven universities that reported having used the SRT, two had used it twice, eight had used it once (with two of these being in the process of using it again or on an ongoing basis) and one did not respond to this or further questions. Of the two universities planning to use the tool, one had already drawn on it and one felt that it would provide benchmarking data and useful sector-wide information.

Data are presented under five subheadings, using the global themes generated through thematic analysis (largely mirroring the areas addressed in the focus group schedule and presented in this order): motivations; process; outcomes/benefits; challenges/suggested improvements; and future use. Each theme is introduced with a summary of key findings (drawing on both the Stage One questionnaire and the Stage Two focus groups). This is followed by a discussion of themes and subthemes (Table 1), using illustrative quotations. These qualitative data are primarily drawn from the Stage Two focus group research, although some quotations from the Stage One questionnaires are included (in parentheses).

### Motivations

The most commonly cited motivations in the Stage One questionnaire were to increase understanding of the whole system approach (nine HEIs – 90%); to create an initial benchmark (nine HEIs – 90%); to inform action planning (seven HEIs – 70%); to measure progress over time (four HEIs – 40%); and to gain stakeholder buy-in (four HEIs – 40%). These themes were echoed and developed in the Stage Two focus groups.

Interest in benchmarking was a key motivator for using the SRT to establish an internal baseline and to assess progress to date and over time, rather than to compare progress with other universities. Respondents felt that, as a national tool endorsed by the UK Healthy Universities Network, the SRT had credibility and could act as a catalyst to others in their HEIs to take action:

*“[The SRT] was really helpful in helping us guide our thoughts and our conversation. But also, I think, in being able to hang our responses on … Having been prompted to think about this by this tool, which is used nationally…I think that it has a bit of kudos, which is important to be able to then take forward discussions, and sometimes arguments.”*

Whilst some had an *a priori* desire to develop or build on a specific area of work, the majority of the HEIs involved in the focus groups already had a Healthy University Steering Group (or equivalent) in place and viewed the SRT as offering a mechanism for structuring self-reflection and undertaking a ‘stock-take’ and action-planning:

*“… it was felt that it was a good thing to do at that time, within the steering group, to get a feel for what perceptions were about how ‘bought in’ the University was around these issues of health and wellbeing.”*

Most respondents cited their engagement with the UK Healthy Universities Network as a key factor motivating them to engage with the SRT. Additionally, for many, a key concern was to strengthen a ‘whole university’ approach, thereby connecting disparate strategies and activities, and more effectively addressing health and wellbeing:

*“We have a long-established health and wellbeing strategy for staff and, to a less structured extent, support and provision for the wellbeing of students. In recent years we have started to work towards a more joined-up approach. The self review tool was useful to review our progress to this end.”*

### Process of completion

The process of completing the SRT took varying lengths of time, with the Stage One questionnaire data showing that four HEIs (40%) took four to eight weeks, three HEIs (30%) took less than one week, two HEIs (20%) took one to four weeks and one HEI used the tool on an ongoing basis. The data also highlighted a range of approaches to engaging with and completing the SRT: undertaken by a number of individuals providing information (four HEIs – 40%); undertaken by one individual (two HEIs – 20%); completed at a stakeholder meeting (two HEIs – 20%); and completed by a lead individual with others contributing (two HEIs – 20%). These issues were developed further in the focus group research as follows.

There was a consensus that, in order to be effective, completion of the SRT had been a cross-university process – engaging multiple stakeholders as active participants. A common approach was to use a Healthy University Steering Group, bringing together senior staff from a range of services, faculties and departments. A number of universities also highlighted the importance of involving their students’ union and external agencies.

The value of bringing diverse stakeholders together to share views and learn from one another was emphasised – as was the necessity of taking a coherent, systematic and thought-through approach to gathering views and ensuring that all stakeholders involved in the process were active contributors. It was noted too that there were sometimes difficulties in answering certain questions, highlighting the importance of engaging across the university:

*“I think some of the feedback we got, when we came back together as a steering group, was that some people struggled with answering some of the questions from particular sections, where they didn’t have much knowledge or expertise.”*

Whilst stressing the need to involve people with sufficiently wide knowledge and expertise, one of the HEIs felt that their comparatively small size, coupled with their decision to establish a small co-ordination group, avoided some of the issues that could arise when managing the SRT process in a larger context. For this honesty to be made manifest, people had to feel comfortable in challenging each other regarding how achievements were self-reported and negotiating a collective position:

*“Separate partners went off and completed the self review tool themselves, and there were a lot of inconsistencies. So some people’s areas came out as a hundred percent (‘we fully met this’). And then when we brought it to the meeting, we kind of had a bit of a debate … And then we kind of drilled down and eventually we worked out, actually, it’s partially met, not fully met.”*

Reflecting on how they undertook the review, many participants saw the role of a Healthy University co-ordinator (or other cross-university lead) as pivotal – not least in terms of communicating the ethos and understanding that underpinned the tool to stakeholders who were less familiar with this thinking. The most frequently used approach was for the Healthy University co-ordinator to introduce the tool to members of the steering group, and for these and other relevant stakeholders to fill in the relevant sections of the downloaded questionnaire. These responses were subsequently returned to the co-ordinator, who collated them and completed the online SRT, generating a ‘traffic light’ report:

*“We briefed everybody and then got them to go through it themselves … And then everyone fed their questionnaires back to me and I pulled it all together, inputted it in to the toolkit and came out with a report at the end.”*

A variation to this approach was for the diverse stakeholders to meet together and discuss and agree answers to the questions. These meetings sometimes took place after the individuals had completed separate versions of the downloaded questionnaire: they then debated the answers and related evidence until they achieved a group consensus, which the co-ordinator inputted online. Another approach was for the Healthy University co-ordinator to introduce the SRT to relevant stakeholders and discuss the content with them individually, prior to completing the online questionnaire using their own existing knowledge supplemented with specialist knowledge drawn from this wider consultation.

Echoing the finding relating to honesty and negotiation, a major challenge experienced by participating universities was the difficulty of ensuring a consistent approach to answering the multiple-choice questions. This highlighted the importance of putting in place an appropriate group process to review individual drafts and debate answers in the light of evidence.

### Outcomes and benefits

The research showed that all HEIs using the SRT had found it to be accessible and easy to use, with the Stage One questionnaire data revealing that six (60%) felt that it was ‘user-friendly’ and four (40%) felt that it was ‘very user-friendly’. The Stage One questionnaire data also profiled a number of benefits of using the SRT, many of which were also emphasized and further explored in the Stage Two focus group research:

- supported understanding of a whole system approach (ten HEIs –100%)
- helped direct future planning (nine HEI’s – 90%)
- facilitated colleagues across the university to work together (nine HEIs – 90%)
- provided a benchmark tool to measure future progress (eight HEIs – 80%)
- brought together a ‘picture’ of work across the university related to the Healthy University (eight HEIs – 80%)
- informed the Healthy University action plan (seven HEIs – 70%)
- helped develop strategic support (five HEIs – 50%)
- encouraged colleagues to become part of the Healthy University (four HEIs – 40%).

Respondents were unanimous in reporting that they had found the SRT to be accessible, user-friendly and appropriate in terms of overall design and content – consequently able to secure the buy-in and participation of multiple stakeholders. Furthermore, the ‘traffic light’ reporting system was described as a clear and engaging way by which to communicate results and ‘next steps’. Moreover, the SRT was widely described as having provided a valuable catalyst, encouraging debate and active participation in the Healthy University process:

*“It really stimulated discussion and gave us a grounding from which to push forwards with it.”*

In particular, Healthy University co-ordinators found it to be useful as a framework for guiding steering group discussions and identifying gaps in knowledge and expertise. It thus served as a springboard for engaging new stakeholders, enhancing inter-departmental and interdisciplinary working, and identifying new champions. In this context, the SRT – in part due to its status as a nationally recognised tool – also prompted appreciation of other people’s work, validated various contributions and enhanced staff worth:

*“I think one of the things that was really helpful about the tool, was that … it did make [people] feel more valued because they saw themselves as part of a national tool, you know. So … they saw that they really did have a relevant part to play. This was really something … it underlined the importance of what we were doing.”*

A key benefit was in enabling stakeholders to review their university and identify areas of strength. Alongside this, it enabled identification of areas where further work and development was needed, helped provide evidence to support arguments for further investment and created a collective space for constructive reflection:

*“It helped us to be able to say, ‘well here are some gaps…some bits we don’t think we’re doing so well and we would like to improve on that’. Obviously, there’s always resource implications if you’re wanting to do something differently, you need evidence to show that more resources are needed. And, you know…that was also a good way of kind of starting a conversation…This is a tool to help us think, and that’s really helpful.”*

*“I think it was actually quite an interesting thought process to go through. What it asked you was questions you didn’t really consider until asked… I think that highlighted across the piece how little, for some of these areas, that we do consider as a University…It’s not the kind of thing that you reflect on, on a daily basis, because you’re busy doing other things.”*

Some respondents reflected that the process of engaging and bringing together diverse stakeholders provided a valuable platform on which to build, through undertaking future planning. The SRT allowed institutions to consolidate existing work, identify gaps and areas of duplication, and clarify future directions in a more coherent and strategic way. In certain instances, this meant using the results as a ‘benchmark’ and motivator for stakeholders to continue good practice and prioritise areas for improvement:

*“It was a really good opportunity for us…just to say ‘well done on all the work that they’ve done’ and to carry on. And then look to how we can improve. [Many of] the things we really wanted to work on, the ‘ambers’, they were kind of minor improvements that we could quite easily implement. And the tool kind of helped us give it that extra push to say, oh, you know, ‘it will help us get on the green if we did this’.”*

Importantly, the SRT was also valuable in enabling Healthy University co-ordinators to engage and secure buy-in from senior level staff. Its nationally recognised status, together with its reporting system, made it attractive to decision-makers – and co-ordinators were able to feedback results of high-level executive groups and committees:

*“It helped us document and evidence the impact and outcomes from our work, which was extremely useful for our annual presentation with the Directorate.” (Questionnaire response)*

Mirroring the motivations for using the SRT, a further perceived benefit was its role in enhancing understanding of Healthy University concepts and, specifically, of whole system working – among steering group members and wider stakeholders. This was the case even in institutions where the Healthy University was well-established and a holistic approach was already perceived to be well-embedded:

*“We have all been working together for a long time. And so I think the benefits of that more holistic approach have been recognised and understood quite well before the tool. I think it has helped, though, to show that everybody’s contribution is really important to the whole. And yes, I think it’s definitely taken people a stage further, in terms of their understanding, even though I think we were quite well advanced beforehand.”*

Alongside this, the process of engaging multiple stakeholders from across the university proved valuable in increasing knowledge and understanding of the contribution of different services and ‘parts of the whole’:

*“[It helped] raise awareness, about the estate’s impact in some of this, you know, that may be not have occurred to us. So it’s an awareness tool as well, it’s quite helpful.”*

Some universities reported tangible developments as a result of completing the SRT – with a distinct shift beyond traditional understandings of health and wellbeing to embrace a whole system ‘Healthy and Sustainable University’ perspective.

### Challenges and suggested improvements

In relation to challenges encountered by universities in using the SRT, the Stage One questionnaire results showed that eight HEIs (80%) reported no negative outcomes, whilst two HEIs (20%) drew attention to specific shortcomings – missing the opportunity to incorporate the student voice; and revealing inconsistencies between the multiple choice answers selected by different stakeholders. The focus group data echoed the first of these points and also highlighted challenges relating to focus, structure and content.

A number of HEIs highlighted that the SRT does not differentiate between staff and student wellbeing. They found this challenging, as it did not allow for responses to reflect their experiences of having performed strongly with one population group and less strongly with another. Consequently, it was felt that the results did not capture more nuanced approaches to developing Healthy Universities work. It was also noted that the overriding focus of the SRT was health and wellbeing as a whole rather than specific health issues and topics. For some, this was considered limiting, as it did not allow differentiation in performance across different areas to be reported:

*“We were quite good at leadership and governance in the food area, but were not so good in terms of sexual health, for instance. So it’s a hard one to just say ‘yes, we cover leadership and governance’ – because for us it really depends on the area…Again, for communication, information and marketing, for instance, we’re very good on things like mental health, food and physical activity, but we can definitely be doing more, in terms of alcohol and drugs…So I think that’s where it was quite hard to complete the tool.”*

It was suggested that the process by which the responses were converted to a percentage rating could usefully be made more explicit and there was also concern that the questionnaire format did not allow questions to remain unanswered or offer a ‘don’t know’ option. It was felt that data gathered from the different universities may thus be skewed, with respondents completing the SRT inaccurately rather than being able to acknowledge a gap in knowledge. Furthermore, not all HEIs were aware of the help and advice available via the wider UK Healthy Universities Network website – and it was suggested that it might be helpful to include links from the SRT to the case studies and other relevant sections.

It was suggested that it might be valuable to include questions designed to explore respondents’ knowledge of and perspectives on Healthy Universities:

*“It would be nice to see a question that measures what their understanding of Healthy Universities actually is…You know, one person could think ‘oh I’ve done a no-smoking day poster’ and that might, for them, fulfil a criteria…Whereas somebody else might have spent six months targeting staff and using occupational health and things like that…”*

Linked to this, one university suggested incorporating questions that aimed to understand underlying motivations and the process of change:

*“What drives people to make the changes…to actually focus on a Healthy Universities approach? And I don’t think, as I recall…there was a question about what drives people, or has driven people, to do what they’ve done…I think that would be interesting…Is it the approach itself or is it actually something that underlies that?”*

Several HEIs raised particular topics that they would like to see strengthened in the SRT – namely health and wellbeing in the curriculum and green space. Additionally, some respondents commented that they had found it difficult to demonstrate innovation and particular areas of excellence when answering the SRT – as the questions tended to compartmentalize activity and not offer this flexibility:

*“We couldn’t quite show everything that we were doing through it, or we couldn’t quite get to…some of the more innovative areas of our work. It couldn’t quite come out through the tool.”*

The suggestion that there could be a qualitative section allowing universities to reflect on their achievements and highlight these was echoed by one questionnaire respondent, who did not use the tool because they found it too prescriptive to engage stakeholders. Related to this issue of flexibility and also to the wider question of ensuring consistency, there was a suggestion that it would be valuable to include an explicit focus on ‘evidence’. Whilst the introduction to the SRT does encourage universities to collate supporting evidence, it was felt that this could be incorporated within the tool itself.

### Future use

Stage One questionnaire data revealed that all ten HEIs (100%) that had already used the SRT and responded to the question would use it again. The Stage Two focus group data explored the potential future use in more detail.

Looking to the future, HEIs highlighted the importance of securing or further strengthening senior-level buy-in, alongside widespread ownership from staff and students.

*“We still want to maintain that top-level position. So we still want those senior people involved. We don’t want to dilute that, but at the same time recognise that we need more of an input from the student body itself.”*

One HEI spoke optimistically of their aim of getting the Vice Chancellor, Deans of Schools, Heads of Units and Students’ Union to work together in a steering and promotion group – and to use the SRT with them:

*“Potentially in the future we’d have a [health and wellbeing] steering group and a sort of promotion group. And we thought that the steering group could do [the SRT] collectively possibly. I think we might probably use it for the steering group, like almost a task for them to do it individually. And then bring it back and complete it altogether, so you get a bit of a better understanding across the whole University.”*

There were some frank discussions about the importance of using the information and data emerging from the SRT process in an effective way to engage senior management and sustain their interest:

*“I think there’s an issue for us here, in how we use this information. And if we’re honest, we haven’t used it as effectively as we might have done…We got this information and we didn’t really know where to take it. Because unless you’ve got senior management involvement and buy in right at the top level, it’s a little bit of a quandary what to do with the information.”*

All HEIs participating in the focus group research said that they would use the SRT again. They acknowledged that using it once provides an internal ‘benchmark’– and that it will only be by using it on a regularly recurring basis that they will be able to monitor and measure progress. A final recognition concerned the importance of universities not becoming complacent, even when they achieve ‘green’ ratings. It was felt that such ratings should lead onto deeper reflection and encourage a culture of continual improvement – a focus that could potentially be incorporated into the SRT.

## DISCUSSION

The research findings suggest that the study fulfilled its goal and aims – scoping its use and impact; examining whether the SRT has supported universities to understand and embed a whole system approach; and informing future developments. Key limitations of the study were the relatively low number of questionnaire respondents and the small number of focus groups. The former perhaps reflects the pressures facing staff within higher education, whilst the latter was in part due to resource constraints and reflects the challenges of securing research funding for the evaluation of Healthy Universities. It is important to acknowledge that the perspectives of non-respondents might well have been different to those of the respondents and reflect less positive views relating to the SRT. However, both stages of the study generated a wealth of informative data, and the analysis revealed a wide and rich range of perspectives concerning motivations; process; outcomes/benefits; challenges/suggested improvements; and future use. Whilst a diversity of themes emerged under each of these global headings, it was noteworthy that there were also cross-cutting insights that permeated the findings.

First, the SRT was well-received and perceived to be beneficial to HEIs engaged in Healthy Universities. Participants highlighted the tool’s accessibility and user-friendliness and largely welcomed the diverse but structured content – articulated in language that a cross-section of people working in HEIs could relate to and engage with. This reflects the wider literature, which argues strongly that a Healthy Universities approach must take account of not only public health but also higher education drivers, “being guided by the distinctive culture and context of universities” (Dooris *et al.*, 2010: 8). To ensure that health and wellbeing are fully embedded, it is necessary to understand how HEIs work to align health with the organisation’s core business and to initiate and manage change (Dooris *et al.*, 2014). There was also evident kudos attached to the status of the SRT, it having been developed and endorsed by the UK Healthy Universities Network as part of a project supported by the Higher Education Funding Council for England (Dooris and Powell, 2012). This kudos was perceived to have been key to securing senior-level buy-in and action.

Second, internal ‘benchmarking’ was found to be both a major motivation for engaging with the SRT and a concrete outcome with several benefits. Whilst the decision to develop a self-assessment process was pragmatic, appreciating the resource-intensive nature of formal accreditation, the findings reinforce results from an earlier consultative research project, which suggested that external assessment could result in an unduly prescriptive approach and emphasised the value of a lighter-touch process-focused approach in securing meaningful organisational change (Dooris *et al.*, 2010). Additionally, there was support for the ‘traffic light’ reporting system, suggesting that this mode of output resonated with users and offered a resource that could be used in a tangible way to communicate findings, catalyse further engagement and inform future planning.

Third, both the content and the process of using the SRT proved useful in building a wider and deeper understanding of the whole system Healthy Universities approach. This emphasis flows from an understanding that whole system thinking is a fundamental characteristic of the healthy settings approach, concerned to secure high-level leadership, engage a wide range of stakeholders, and combine high visibility health-related projects with system-level organisation development and the implementation of multiple interconnected interventions and programmes (Dooris, 2013; Shareck *et al.*, 2013).

Fourth, the experience of undertaking the review proved to be enormously valuable in catalysing and strengthening cross-university and cross-sectoral partnership and collaboration. As argued by Dooris (2006), mapping and connecting diverse groups of stakeholders from within and beyond the university forms a key aspect of the Healthy Universities approach – and clearly facilitates the strengthening of system-wide working. Furthermore, the importance of facilitating the student voice was highlighted, a finding that resonates with other research and policy (Holt *et al.*, 2015; Trowler, 2010).

Fifth, whilst the findings were overwhelmingly positive, areas for potential enhancement were identified, including:

- distinguishing between performance relating to work with students and staff
- separating out action relating to different health-related themes and issues
- strengthening the focus on academic development and the integration of health and wellbeing into curricula
- encouraging universities to incorporate student perspectives
- including a more explicit means of capturing evidence to support responses
- prioritising the capture of innovation and creative practice
- embedding a focus on continual improvement.

## CONCLUSION

The Healthy Universities approach is gaining momentum worldwide, as evidenced by the growing number of national networks, the participation of 375 participants from over 30 different countries in the 2015 International Conference on Health Promoting Universities and Colleges, and the launch of the Okanagan Charter for Health Promoting Universities and Colleges (2015, p.2) – which sets out a radical and far-reaching vision:

“Health Promoting Universities and Colleges transform the health and sustainability of our current and future societies, strengthen communities and contribute to the wellbeing of people, places and the planet…[They] infuse health into everyday operations, business practices and academic mandates. By doing so…[they] enhance the success of our institutions; create campus cultures of compassion, wellbeing, equity and social justice; improve the health of the people who live, learn, work, play and love on our campuses; and strengthen the ecological, social and economic sustainability of our communities and wider society.”

Despite these encouraging signs, it is also clear that universities face continuing challenges in securing resources for developing, implementing and evaluating this important area of work, perhaps because – alongside a widespread perception that university populations are privileged and therefore not a priority focus for investment of health promotion resources – explanatory theory for the settings approach as a whole remains underdeveloped and there is a scarcity of evidence for Health Universities in particular. (Newton *et al.*,2016). Looking to the future, it is clear that HEIs seeking to implement the Healthy Universities approach will need tools and frameworks that offer them real utility. The research study found the SRT to be an enormously valuable tool which, when engaged with fully, offered significant benefits to HEIs seeking to improve the health and wellbeing of their communities. These benefits were felt by HEIs at different stages in the Healthy Universities ‘journey’ and spanned outcome and process dimensions: not only did the SRT generate an easy-to-understand report summarizing strengths and weaknesses; it also served as a catalyst to effective and whole system partnership working. Moreover, the potential enhancements identified through the research offer opportunities to increase the tool’s utility further.

Whilst the UK Network, its website and the SRT exist primarily to support UK universities, they have proved attractive to universities across the world: the Network has 16 associate members from six countries spanning four continents; since its launch, the website has had visitors from 150 countries; and over 30 universities from outside the UK have now registered to use the SRT. Building on the momentum created by the 2015 International Conference, the Network is now working with international colleagues to create a truly global movement for Healthy Universities.
